# A Practical Guide for the Quality Evaluation of Fluobodies/Chromobodies

**DOI:** 10.3390/biom14050587

**Published:** 2024-05-15

**Authors:** Urša Štrancar, Claudia D’Ercole, Lucia Cikatricisová, Mirna Nakić, Matteo De March, Ario de Marco

**Affiliations:** Laboratory of Environmental and Life Sciences, University of Nova Gorica, Vipavska cesta 13, Rožna Dolina, 5000 Nova Gorica, Slovenia; ursa.strancar@ung.si (U.Š.); claudia.dercole@ung.si (C.D.); lucia.cikatricisova@ung.si (L.C.); mirna.nakic@ung.si (M.N.); matteo.demarch@ung.si (M.D.M.)

**Keywords:** fluorescent proteins, fluobodies, protein degradation, alternative start codons

## Abstract

Background: Fluorescent proteins (FPs) are pivotal reagents for flow cytometry analysis or fluorescent microscopy. A new generation of immunoreagents (fluobodies/chromobodies) has been developed by fusing recombinant nanobodies to FPs. Methods: We analyzed the quality of such biomolecules by a combination of gel filtration and SDS-PAGE to identify artefacts due to aggregation or material degradation. Results: In the SDS-PAGE run, unexpected bands corresponding to separate fluobodies were evidenced and characterized as either degradation products or artefacts that systematically resulted in the presence of specific FPs and some experimental conditions. The elimination of N-terminal methionine from FPs did not impair the appearance of FP fragments, whereas the stability and migration characteristics of some FP constructs were strongly affected by heating in loading buffer, which is a step samples undergo before electrophoretic separation. Conclusions: In this work, we provide explanations for some odd results observed during the quality control of fluobodies and summarize practical suggestions for the choice of the most convenient FPs to fuse to antibody fragments.

## 1. Introduction

The production of fusion constructs formed by a recombinant binder and a fluorescent protein (FP), often called chromobodies or fluobodies, represents an extremely convenient and inexpensive way to recover reagents suitable for applications such as flow cytometry or fluorescent microscopy [1,2,3,4]. Information relative to the sequences and functional characteristics of FPs is available (https://www.fpbase.org/) and, therefore, the design of a set of expression vectors differing for the sequences encoding FPs can be conceived with the aim of generating fluorescent immunoreagents suitable for multidimensional analyses [5]. The physical link connecting the binder and the FP guarantees a 1:1 ratio between the two components of the reagent. Furthermore, site-specific labeling prevents modifications that can affect the structure and functionality of the binder paratope. However, the quality of the obtained reagents must be controlled to avoid artefacts due to aggregation or material degradation. In our lab, we express fusions between FPs and either nanobodies or adhirons in the cytoplasm of BL21(DE3) bacteria co-expressing sulfhydryl oxidase (SOX), a condition that allows for the correct folding and functionality of both components [6]. Nevertheless, previously published data reported that some proteins, such as mCherry, despite being strongly monomeric, showed a propensity to aggregate when fused to some partners, whereas the SDS-PAGE analysis of other FP constructs evidenced an apparent degradation of the chimeras [7]. We noticed that this latter process was common in the presence of different FPs. Despite frequent informal reports from other labs confirming this observation, the scientific community has not dedicated much effort to elucidating this issue, since it is usually considered a sort of routine drawback of the protein production process and not a consequence of FP characteristics. 

The data available in the literature suggest three possible explanations for the odd migration of FP fusion constructs in observed SDS-PAGE. The first is that the FP moiety is internally cleaved after having reached its native folding, which is what happens in FusionRed-derived constructs [8]. The cleavage does not affect the fluorescent efficiency of the FP but, after denaturation, three polypeptides are separated by electrophoresis [7]. The second explanation is that more expression products can be generated from the same mRNA because its sequence might be compatible with the selection of alternative start codons [9]. In eukaryotic systems, it is known that the binding of regulative proteins on upstream open reading frame sequences constitutes a modality for shifting the start codon and obtaining alternative protein synthesis [10]. In prokaryotes, the relevance of alternative start codons has been underestimated and their annotation has often been misreported or completely omitted [11,12], but it has recently been demonstrated that sub-optimal start codons, such as GUG and UUG, can be activated with mechanisms that can require compensatory mutations in the Shine–Dalgarno sequence [13,14]. The third hypothesis does not consider biological reasons but focuses on trivial technical aspects that could induce protein degradation, as in the case of mCherry, during either expression/purification or sample preparation and separation [7,15]. To summarize, the presence of multiple bands corresponding to FP constructs might be the result of alternative synthesis or of full-length protein degradation, either in vivo or during sample manipulation. The specific case of self-cleavage typical of the FusionRed lineage has been overcome by means of rational mutagenesis that resulted in more stable variants [7]. In this work, we used model constructs to elucidate what happens to FPs of different origin with the aim of providing suggestions for their choice and practical analytical solutions to all researchers who need to characterize their FP-based reagents. Furthermore, the different monomeric isoforms of the recently described green FP StayGold [16] were assessed for their capacity to produce effective immunoreagents when expressed in bacteria, since it is helpful to have access to alternative FPs possessing different features in terms of polymerization state, brightness, and both photo- and structural stability.

## 2. Materials and Methods

### 2.1. Construct Cloning

Synthetic genes for the fluorescent proteins StayGoldE138D [17], mStayGold QC2-6 FIQ [18], and mBaoJin [19] were ordered from Twist Bioscience (San Francisco, CA, USA). The DNA sequence for mStayGoldE138D was inferred from the available amino acid sequence and optimized for *Escherichia coli* expression by applying the Codon Optimization Tool available on the Twist Bioscience website. The published sequences were used for mStayGold and mBaoJin. All three genes were cloned into an adapted pETM11 vector in which the TEV cleavage site was deleted and a 6xHis tag was present at the N-term. Primers ordered from Kemomed d.o.o. and repliQa HiFi ToughMix^®^ (Quantabio, Beverly, MA, USA) were used for both insert and vector amplification. Amplified sequences were separated by agarose gel electrophoresis (1% *w*/*v* agarose) and cleaned using a peqGOLD Gel Extraction Kit (PEQLAB Biotechnologie, Erlangen, Germany). An insert-to-vector ratio of 3:1 was used and mixed 1:1 with Gene Art™ Gibson Assembly MasterMix (Invitrogen, Waltham, MA, USA). After 20 min of incubation at 50 °C, 60 μL of competent DH5α *E. coli* cells was transformed with 5 μL of the Gibson Assembly Mix. The transformed cells were plated on Luria–Bertani (LB) agar 1.6% (*w*/*v*) dishes containing 50 μM kanamycin and were incubated overnight. Three colonies were grown separately in 5 mL LB media (50 μM kanamycin) overnight. The construct was isolated using peqGOLD Plasmid Miniprep Kit II (PEQLAB Biotechnologie, Erlangen, Germany) and confirmed by Sanger sequencing (Azenta Life Sciences, Burlington, MA, USA). 

The fusion constructs formed by nanobodies (Nbs), fluorescent proteins (mRuby3, mClover3, Electra, mBlueberry2, hyperfolder YFP), and a C-terminal 6xHis tag were cloned as described above and expressed using a modified pET14b vector [20]. The transformed cells were selected on LB plates containing 100 µg/mL of ampicillin and the sequences were confirmed by Sanger sequencing. The tdTomato and Adh-mCherry (Adh is for adhiron) constructs were expressed with a N-terminal 6xHis tag from a pET28a vector.

### 2.2. Protein Purification and Characterization

StayGoldE138D, mStayGold QC2-6 FIQ, mBaoJin, Adh-mCherry, and tdTomato were expressed in BL21 (DE3), while nanobodies fused to the other fluorescent proteins were expressed in BL21(DE3) co-expressing a sulfhydryl oxidase (SOX) to favor the formation of disulfide bonds [6]. Bacteria were grown in 500 mL of LB media (50 μM kanamycin) at 37 °C and 210 rpm. When the wild-type bacterial culture OD_600_ reached 0.6, expression was induced with 0.1 mM IPTG, and the temperature was decreased to 20 °C for the overnight growing. SOX cells were grown in 1 L of LB media (100 μg/mL ampicillin, 34 μg/mL chloramphenicol) at 37 °C and 210 rpm; 0.2% (*w*/*v*) of arabinose was added at the OD_600_ of 0.4 to induce SOX expression. The temperature was decreased to 20 °C and fluobody expression was triggered after a further 30 min (OD_600_ around 0.6) by adding 0.2 mM IPTG. After overnight incubation, both wild-type BL21 (DE3) and SOX cells were harvested by centrifugation (4500× *g* for 30 min) and resuspended in 20 mL of 50 mM Tris-HCl pH 8.5, 500 mM NaCl, and 5 mM MgCl_2_. After three cycles of freezing and thawing, lysozyme (100 μg/mL) and DNase I (33 U/mL) were applied to the lysate and after 30 min of incubation at room temperature, the lysates were sonicated and centrifuged at 13,000× *g* for 30 min at 4 °C. Proteins were isolated using a Talon Hi-Trap column (Cytiva—Marlborough, MA, USA) equilibrated with 50 mM Tris-HCl, pH 8.5, 500 mM NaCl, and 15 mM imidazole controlled by a ÄKTA pure™ system. After washing with 50 mM Tris-HCl, pH 8.5, 500 mM NaCl, and 15 mM imidazole, the proteins were eluted in the same buffer but using 150 mM imidazole. The samples were immediately desalted using a Hi-Trap Desalting column (Cytiva) equilibrated with PBS/5% glycerol, pH 7.4. Samples of purified proteins were evaluated by SDS-PAGE and gel filtration. In the case of SDS-PAGE, samples (30 µL) were mixed 4:1 with 4× loading buffer (2 mL Tris-HCl pH 6.8, 0.8 g SDS, 2 mL glycerol, 0.4 mL 2-mercaptoethanol, 1 mL EDTA 0.5 M, 8 mg bromophenol blue in a final volume of 10 mL) and either boiled for 5 min at 95 °C or directly loaded on the polyacrylamide gel. After a run performed at 21 °C, gels were stained with Coomassie brilliant blue and destained with a solution of 40% methanol, 10% acetic acid, and 50% water. Analytical gel filtration was performed using a Superdex™ 75 Increase 5/150 GL column at 21 °C (Cytiva—Marlborough, MA, USA). The column was equilibrated with PBS at pH 7.4 and 50 µL of each sample was injected, exploiting a 50 µL loop, at a flow rate of 1 mL/min. Protein concentration was calculated by applying the specific mass extinction coefficient to the values of UV absorption recorded at 280 nm.

## 3. Results

**Figure 1:**
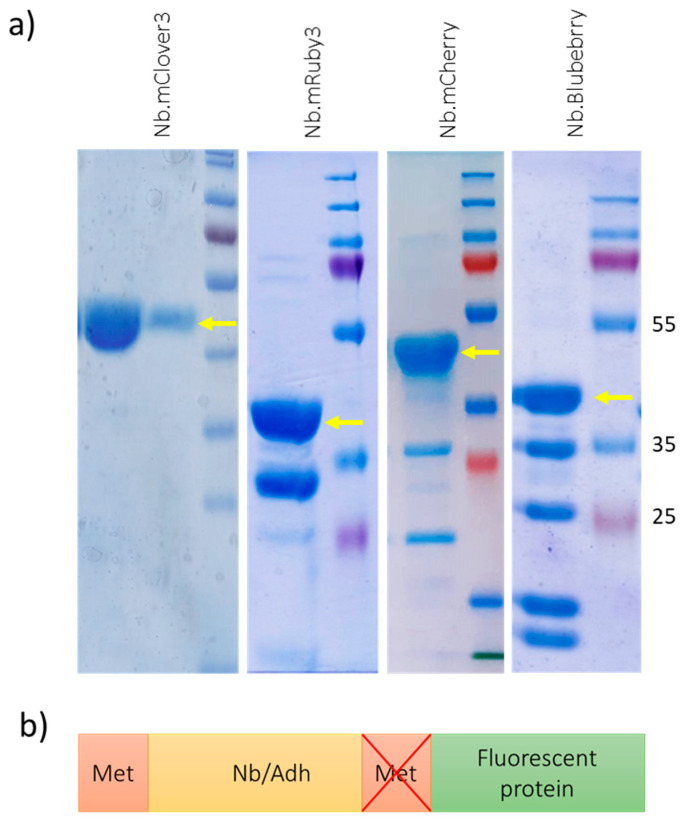
Commonly observed distribution patterns of binder–FP constructs separated by SDS-PAGE. (**a**) FPs belonging to different lineages and fused to recombinant adhirons/nanobodies as they appear after having been boiled in loading buffer and separated by SDS-PAGE. Full-length constructs are indicated by yellow arrows. (**b**) Schematic representation of the nanobody–FP fusion constructs. Cloned FPs either preserved their original N-term starting methionine or this amino acid was removed from the sequence. Original Western blot images are provided in Supplementary Materials.

FPs are pivotal reagents for biological applications and, consequently, there is a constant search for ever-better-performing variants suitable for different applications. In this experiment, we designed vectors for the fusion of nanobodies and adhirons with different FPs. Despite the successful exploitation of such immunoreagents in several kinds of experiments, their quality control often gave odd results; multiple bands corresponding to peptides shorter than the expected full-length construct were detected. Among the examples reported in Figure 1a, only the fusion between Nb and mClover3 showed a single band corresponding to the correct mass. 

When other FPs were used, multiple bands corresponding to shorter constructs appeared after SDS-PAGE together with a band of the expected mass. To rule out the hypothesis that the apparent degradation bands were indeed shorter/incomplete constructs synthetized starting from internal methionines, we compared the SDS-PAGE profiles of constructs in which the FP sequences, cloned at the C-term of the binder (Figure 1b), either did or did not preserve the codon corresponding to their initial methionine. Since no difference was observed, this hypothesis was abandoned, and we focused on purification protocols suitable to eliminate the degradation products.

The gel filtration experiment performed with the nanobody–mRuby3 construct was compatible with a sample composed of both full-length and shorter fragments, even though the boiling step seemed to accelerate the construct’s degradation (Figure S1). We recovered the gel filtration peak corresponding to the full-length nanobody–mRuby3 fusion protein and separated it by SDS-PAGE. Surprisingly, instead of the expected single band, the same pattern of band distribution obtained using the initial sample was reproduced (Figure S1). Consequently, we reasoned that construct degradation might happen during electrophoresis as a consequence of the heating step during sample preparation, whereas the construct could be still mostly intact before SDS-PAGE. On the other hand, since not all the FPs were similarly fragile after undergoing the same preparation protocol (Figure 1a), a specific combination of FPs and experimental conditions had to be considered, and we analyzed different factors. In preliminary experiments, we used several binders (either nanobodies or adhirons with different sequences) but did not see any difference and therefore excluded the possibility that the nature of the fused binding moiety affected chimera stability. Next, we turned our attention to FPs, and those investigated in our experiments were grouped according to their biological origin with the aim of identifying possible correlations between lineages and apparent stability. This information is summarized in Table 1. In the literature, it is reported that red fluorescent proteins derived from different ancestors, such as mCherry and mKate2, are prone to hydrolyzation when denatured and boiled to be separated by SDS-PAGE [7,15]. A similar pattern was also observed in the case of the blue fluorescent protein mBlueberry2, which shares the same ancestor (dsRed from *Discosoma* spp.) with mCherry (Figure 1a). The red fluorescent protein tdTomato belongs to the same lineage of mCherry and mBlueberry2 and seems similarly sensitive to hydrolysis (Figure S2) despite, in contrast to mCherry and mBlueberry2, having conserved its ancestor’s propensity to dimerize [21], as the gel filtration data clearly show (Figure S2). This observation suggests that instability, in this specific case of dsRed derivatives, is not necessary a trade-off of the mutagenesis process performed to obtain monomeric versions of the original polymeric fluorescent proteins.

**Figure 2:**
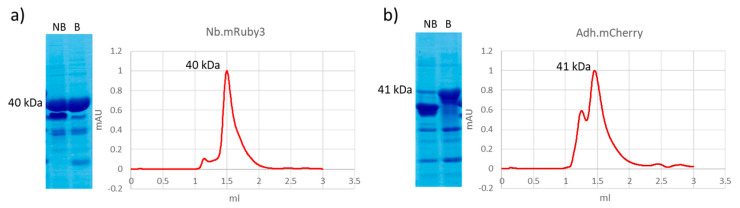
Protein quality control of immunoreagents formed by binders and red fluorescent proteins. Constructs formed by a nanobody fused to mRuby3 (**a**) and an adhiron fused to mCherry (**b**) were analyzed after purification by analytical gel filtration and SDS-PAGE. Both boiled (B) and non-boiled (NB) samples were separated electrophoretically to assess the effect of heating denaturation. Original Western blot images are provided in Supplementary Materials.

Further conditions were assessed to investigate the origin of hydrolysis of sensitive FPs. Apart from some minor differences, the SDS-PAGE distribution patterns of Nb or Adh constructs fused to the monomeric FPs mCherry, mRuby3, and mBlueberry2 were similar for both boiled and non-boiled samples (Figure 2 and Figure S3). Both showed the presence of multiple bands but, when the native Nb.mRuby3 samples underwent gel filtration, we noticed the expected peak of the monomeric construct with only a minor peak corresponding to larger contaminants (compatible with the minor bands of high molecular weight observed in the SDS-PAGE). The Adh.mCherry samples showed a profile with a double peak, the first compatible with the presence of a dimer and the second of the mass corresponding to the monomer, partially tailing and therefore indicating the possible presence of degradation species of lower mass (Figure 2). Altogether, these data indicated that mCherry hydrolysis is probably independent of the harsh denaturation conditions used to prepare SDS-PAGE samples and that it also has a residual tendency to polymerize, as already reported [22]. 

**Figure 3:**
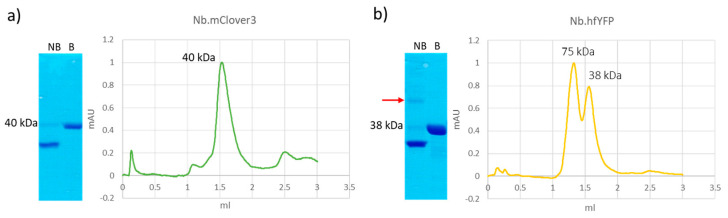
Protein quality control of nanobody immunoreagents fused to fluorescent proteins originating from avGFP. (**a**) Data corresponding to Nb.mClover3; (**b**) Data corresponding to Nb.hfYFP. Constructs formed by a nanobody fused to either mClover3 or sfYFP were analyzed after purification by analytical gel filtration and SDS-PAGE. Both boiled (B) and non-boiled (NB) samples were separated electrophoretically. The red arrow indicates the dimer of the Nb.sfYFP construct. Original Western blot images are provided in Supplementary Materials.

Green FPs derived from avGFP (mEGFP and mClover3) show negligible degradation after undergoing electrophoresis (Figure S4). Next, we characterized the stability of chimera formed by mClover3 and another FP of the same lineage, hfYFP, and nanobodies. The data presented in Figure 3 show that both constructs had no apparent degradation, independently of whether they were separated after heating denaturation (fractions B—boiled) or in the absence of this step (NB—non-boiled). Denatured samples produced a single band of the expected mass (40 kDa) in SDS-PAGE, whereas native-like samples (NB) migrated faster, an effect probably due to different interaction patterns with SDS. In the case of the hfYFP construct, a larger band (red arrow) was visible, and the gel filtration profile confirmed the presence of a dimer (75 kDa) that should represent the prevalent structural form of this FP, according to the available characterization data reported in the literature [23]. In contrast, a single regular peak, corresponding to a monomer, was obtained in the nanobody’s fusion with mClover3. This result evidenced the difference in stability between these FPs and the red ones evaluated above.

**Figure 4:**
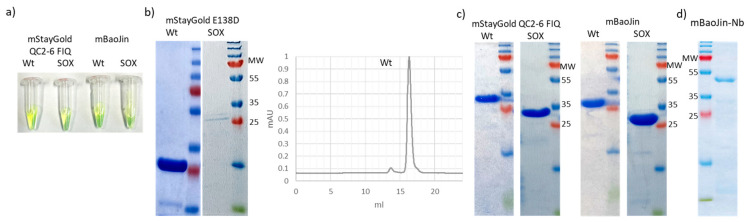
Protein quality control of mStayGold variants and derived fusion constructs formed by nanobody and mBaoJin. Effect of oxidative conditions on FP stability: single variants were expressed in the cytoplasm of wild-type BL21(DE3) bacteria and in the cytoplasm of the same bacterial strain but co-expressing sulfhydryl oxidase (SOX). (**a**) Normalized amounts of proteins (QC2-6 FIQ and mBaoJin expressed in wild-type BL21(DE3) as well as in SOX bacteria) were compared for qualitative evaluation of their fluorescence. (**b**) SDS-PAGE and gel filtration of the E138D isoform. (**c**) SDS-PAGE of the isoforms QC2-6 FIQ and mBaoJin expressed in SOX as well as in wild-type BL21(DE3) bacteria. (**d**) The monovalent StayGold isoform mBaoJin was fused to a nanobody and the resulting purified fluorescent immunoreagent was characterized by SDS-PAGE. Original Western blot images are provided in Supplementary Materials.

In a successive step, we applied the accumulated expertise to characterize the new promising green FP StayGold isolated from *Cytaeis uchidae* (Table 1). The dimeric form of StayGold has recently been described and has exceptional characteristics of stability and brightness [16], which have stimulated the research community to look for monovalent variants. This commitment resulted in the description of three different monomeric proteins ([17,18,19], Figure S5). We produced them in wild-type BL21(DE3) and compared their electrophoretic profiles (Figure 4). All three samples were fluorescent and, when separated by SDS-PAGE, they showed a single band. Minimal hydrolysis products were detectable only in E138D (Figure 4b,c). The monomeric condition was confirmed by the results of gel filtration, which showed a unique, narrow peak that had a small shoulder only, corresponding to the degradation products in the case of E138D (Figure 4b).

Finally, we planned to produce fusions of monomeric StayGold with nanobodies. Nanobody folding depends on the formation of internal disulfide bond(s), which is obtained either in oxidizing compartments, such as the bacterial periplasm, or in the cytoplasm in the presence of oxidizing conditions. Since most FPs do not fold correctly under oxidative conditions, SOX bacteria, which co-express sulfhydryl oxidase and are already able to produce several FP-fusion immunoreagents [6], seemed to be the logical option for producing functional chimera between nanobodies and StayGold in their cytoplasm. We performed a preliminary test by expressing the three monomeric isoforms in SOX bacteria to evaluate any potential interference with FP folding. Unexpectedly, while the two isoforms known as mStayGold QC2-6 FIQ and mBaoJin were expressed at high yields and were fluorescent (Figure 4a,c), the E138D isoform did not preserve its stability and fluorescence, making the production of functional fusion constructs between this FP and nanobodies improbable. Therefore, as a proof-of-principle, we used one of the two compatible StayGold isoforms to prepare a fluorescent immunoreagent in SOX bacteria. The Nb-mBaoJin fusion was successfully expressed and purified as a soluble, fluorescent protein (Figure 4d).

## 4. Discussion

FPs have become indispensable reagents in biological research and, consequently, the scientific community constantly tries to improve their photophysical properties as well as their structural characteristics (valency, stability, monodispersity). The accumulated information suggests that there is no single optimal FP, but rather that several FPs are suitable for alternative applications which require either monomeric or oligomeric forms, different wavelength spectra, and a variety of other chemical features [24]. However, some characteristics remain drawbacks under any circumstance. For instance, many FPs are sensitive to degradation [25,26] and, from this perspective, the efforts to identify not-cleavable mutants of FusionRed, a protein that otherwise is not toxic and does not aggregate, should be appreciated [15]. Furthermore, and despite some exceptions [27], most FPs are sensitive to redox conditions and lose their fluorescence in oxidative environments/cellular compartments regardless of the number of cysteines present in their sequence [28]. This impairment becomes particularly limiting when producing chimera immunoreagents formed by a FP and an antibody fragment. Even the smallest functional construct among them, the single-domain nanobody, possesses at least a disulfide bond and, even though in some cases nanobodies can reach their native conformation in the absence of such a bond, the majority require an oxidative environment to fold correctly. This condition, therefore, would prevent the expression of recombinant fusions between FPs and nanobodies because the two chimera components can exclusively fold only in a cell compartment that is not suitable for the partner. We succeeded in circumventing this shortcoming by expressing a chimera of EGFP and nanobodies in the cytoplasm of bacteria that had been co-transformed to produce a sulfhydryl oxidase and a disulfide isomerase [6]. Successively, we demonstrated that the method was robust enough to produce nanobodies fused to Fc domains as well as to several other proteins and FPs (Figure 1, Figure 2, Figure 3 and Figure 4) [20,29]. Nevertheless, the universality of this method has been now challenged by the evidence that some FPs, such as the monomeric version of StayGold E138D, might lose their fluorescence when expressed under these conditions. In a recent comparative work, this variant showed the lowest brightness when expressed in mammalian cells [30]. The reasons for its sensitivity to the co-expression of sulfhydryl oxidase are not evident but, as indicated in Figure S5, its structure exposes an extra cysteine on the surface that might be involved in the formation of disruptive disulfide bonds during folding, resulting in the impossibility of reaching its functional native conformation. This observation confirms the need to consider all photo-structural characteristics of an FP before choosing it for a specific experiment or to consider, as an alternative, either intrabodies that do not require the formations of disulfide bonds or scaffolds alternative to antibody fragments and without cysteines in their sequence. Whereas intrabodies can be difficult to identify, we recently demonstrated that it is not only feasible, but even straightforward, to design and produce functional immune-like reagents using adhirons isolated by a phage display library [31]. 

The data collected in this contribution suggest that, at least for the preparation of fusion constructs between nanobodies and FPs, the most reliable fluorescent partners are those derived from *Aequorea victoria* (EGFP, mClover3, hfYFP). The monomeric StayGold-mutants mStayGold QC2-6 FIQ and mBaoJin from *Cytaeis uchidae* seem to be valid alternatives to the established green fluorescent proteins because of their extremely elevated brightness. Furthermore, mStayGold QC2-6 FIQ is also extremely photostable, while mBaoJin possesses extremely rapid folding, chemical stability, and resistance to fixatives [19,30]. The group which characterized these FPs is confident they can obtain at least blue and cyan derivatives of mStayGold and we anticipate that mTurquoise2ox (another derivative of avEGFP able to fold at oxidizing conditions) might also be a valid (cyan) alternative to the (deceiving) blue FPs tested in this work for producing fusions with nanobodies. Among the red proteins, mRuby3 appears promising, since our experiments showed that most of the bands observed in SDS-PAGE are only artefacts due to the preparation of electrophoresis samples and do not correspond to real protein degradation. Other options might derive from the near-infrared monovalent variants (miRFP family) obtained from FPs of the bacterium *Rhodopseudomonas palustris* [32], which is easily expressed in *E. coli*, including in combination with nanobodies [4]. Unluckily, the original papers do not describe the protocol used for fusion construct production in detail, preventing a direct comparison with chimera obtained using other FPs. 

## 5. Conclusions

The production of reliable FPs and FP-based immunoreagents is critical to undertaking several downstream biological experiments. Here, we reported on the appearance of artefacts during protein quality analysis that can result in a misleading evaluation of the reagents. Furthermore, a survey of several established and new FPs allowed for the identification of a set of FPs suitable for designing fusion constructs in combination with single-domain binders that can be used for direct antigen detection. 

## Figures and Tables

**Table 1:** Relationship between protein origin and their stability after separation by SDS-PAGE.

Original Fluorescent Protein	Derived Proteins	Degradation Products in SDS-PAGE
eqFP611 (*Entacmaea quadricolor*)	mRuby3, Electra	Yes
avGFP (*Aequorea victoria*)	mClover3, hfYFP, mEGFP	No
dsRed (*Discosoma* spp.)	mCherry, tdTomato, mBlueberry2	Yes
CU17s (*Cytaeis uchidae*)	StayGold, monomeric StayGold variants	No

## Data Availability

There are no further experimental data to share, but the authors will provide the reagents used for research purposes.

## Supplementary Materials

The following supporting information can be downloaded at: https://www.mdpi.com/article/10.3390/biom14050587/s1, Figure S1: Characterization of the fusion construct Nb.mRuby3; Figure S2: Characterization of tdTomato; Figure S3: SDS-PAGE of a fusion immunoreagent composed of a nanobody and mBlueberry2; Figure S4: SDS-PAGE of purified green fluorescent proteins; Figure S5: Alignment of the three monomeric isoforms of StayGold [33,34,35].
