# Supplementary material for: A Practical Guide for the Quality Evaluation of Fluobodies/Chromobodies
Source: Biomolecules. 2024 May 15;14(5):587. doi: 10.3390/biom14050587 (PMC11117837; doi:10.3390/biom14050587)

Supplementary Material

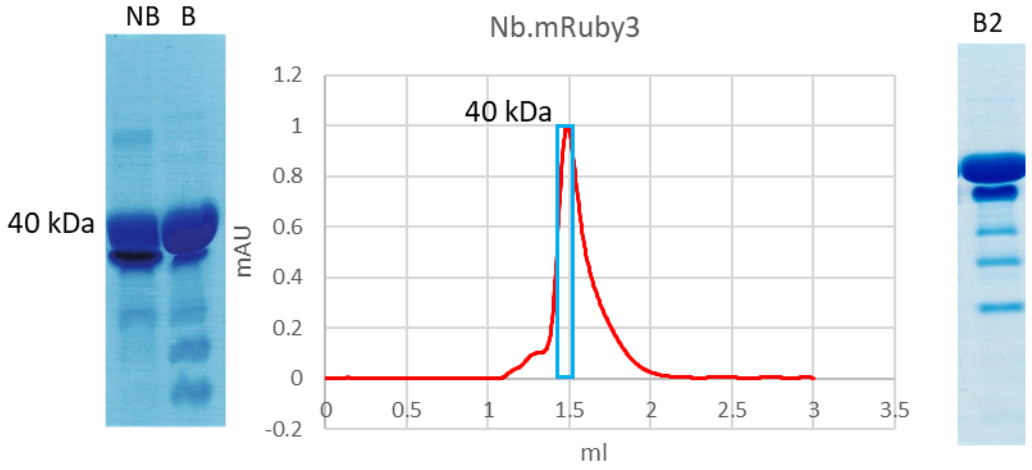

Figure S1. Characterization of the fusion construct Nb.mRuby3

The chimeric protein was analysed by SDS-PAGE, using not-boiled (NB) and boiled (B) samples, and by analytical gel filtration. The elution fraction corresponding to the peak (blue mask) was recovered, boiled and separated again by SDS-PAGE (B2).

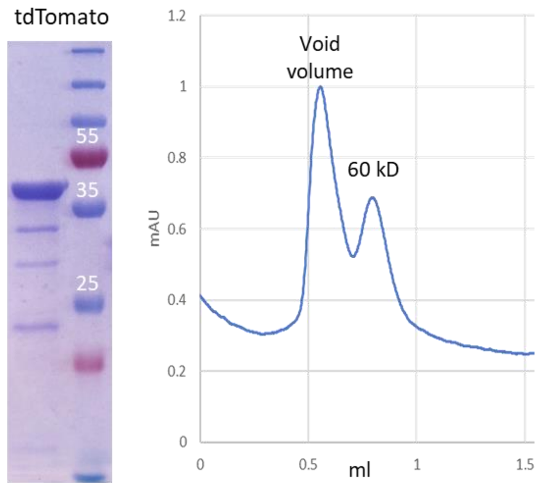

Figure S2. Characterization of tdTomato

The sample was separated by SDS-PAGE, minor degradation bands appeared together the full-length construct. The gel-filtration profile showed the presence of polymerization species (void volume) together with the monomeric fraction of the tandem construct.

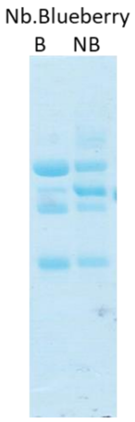

Figure S3. SDS-PAGE of a fusion immunoreagent composed by a nanobody and mBlueberry2

The sample was either boiled (B) or not boiled (NB) before being loaded on the gel and separated by electrophoresis.

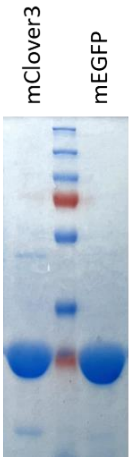

Figure S4. SDS-PAGE of purified green fluorescent proteins

Purified mClover3 and mEGFP were separated by SDS-PAGE.

a)

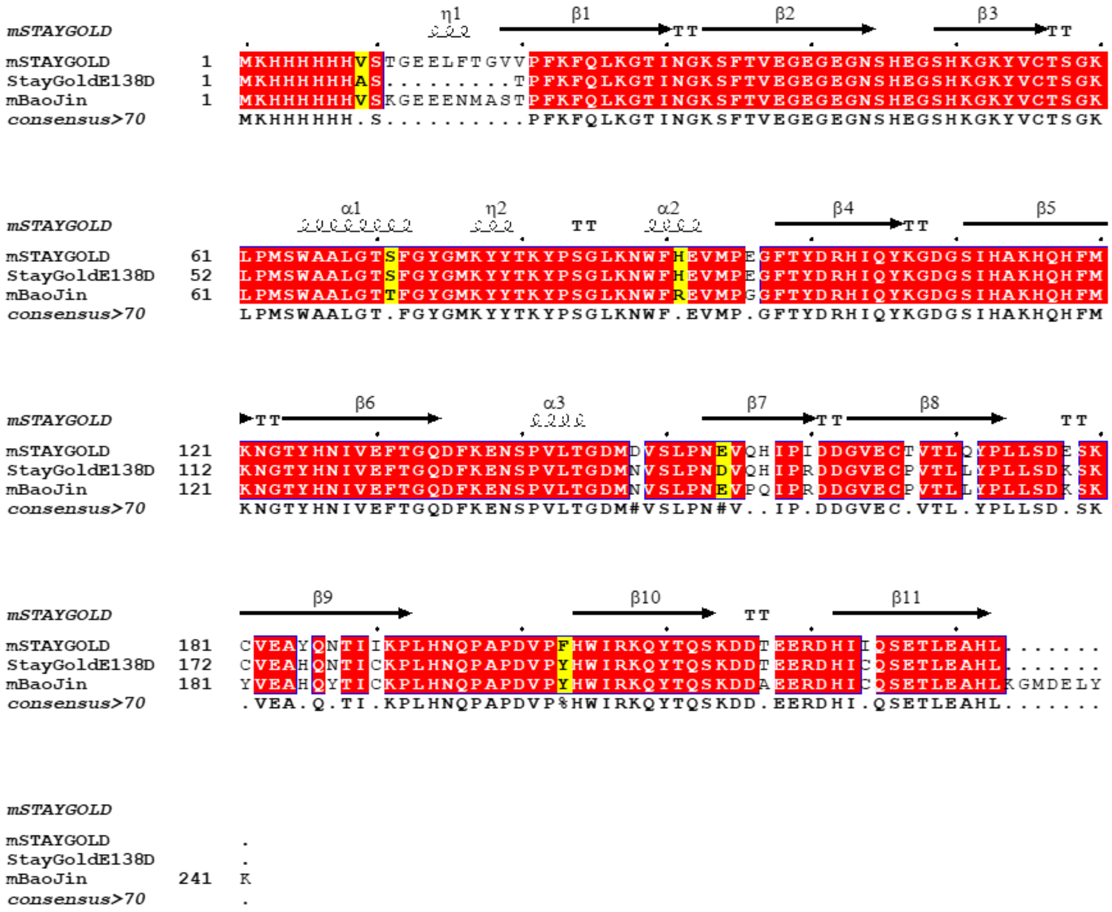

b)

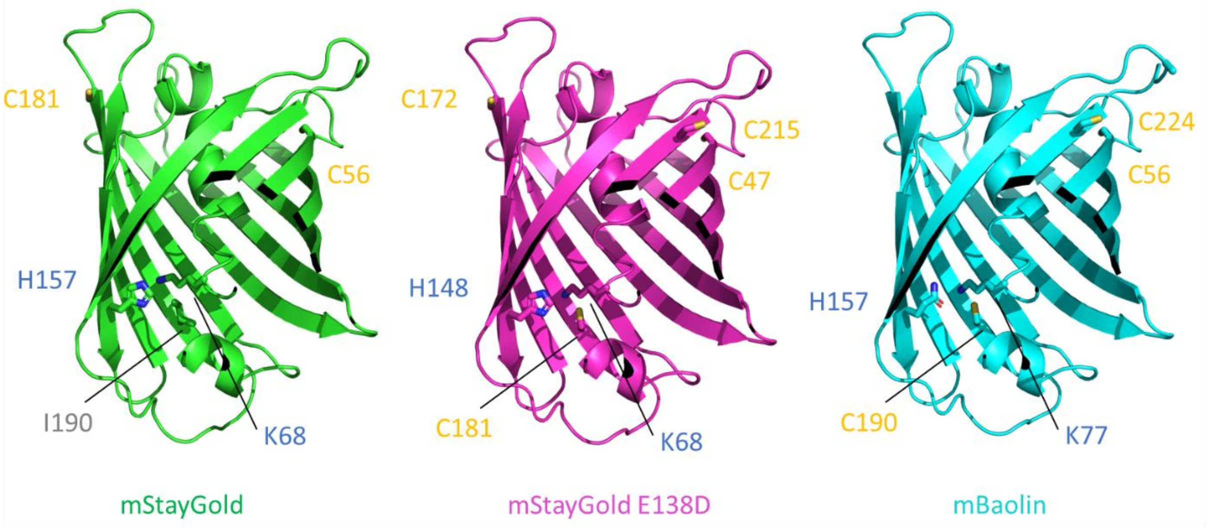

Figure S5. Alignment and structure of the three monomeric isoforms of StayGold

a) Sequence alignment of mStayGold, mStayGold_E138D and mBaolin. b) Structural comparison among mStayGold (green), mStayGold_A138D (magenta) and mBaolin (cyan) visualized in PyMol. Cys residues are indicated in yellow, positively (Lys) or partially-positively (His, Gln) charged residues are in blue, hydrophobic (Ile) in grey. Numbers refer to the snapgene sequences. Sequence alignment of mStayGold, mStayGold_E138D and mBaolin was obtained using PROMALS3D [1] and visualized with ESPript3.0 [2]. mStayGold structures were visualized in PyMol [3].

1. Pei, J.; Grishin, N.V. PROMALS3D: multiple protein sequence alignment enhanced with evolutionary and three-dimensional structural information. *Methods Mol Biol* **2014**,*1079*, 263-71.

2. Robert, X.; Gouet, P. Deciphering key features in protein structures with the new ENDscript server. *Nucleic Acids Res* **2014**, *42*, W320-4.

3. DeLano, W.L. The PyMOL Molecular Graphics System. Delano Scientific, **2002**, San Carlos.

Original images:

Figure 1

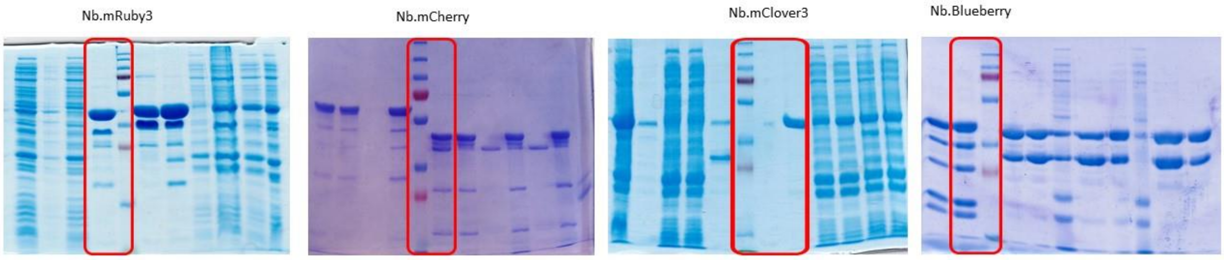

Figure 2

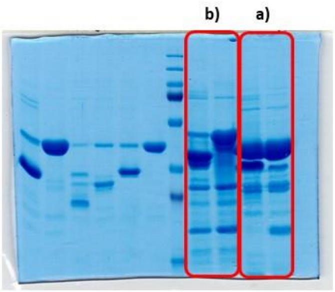

Figure 3

a) **b)**

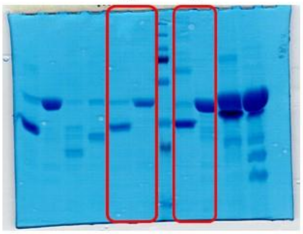

Figure 4

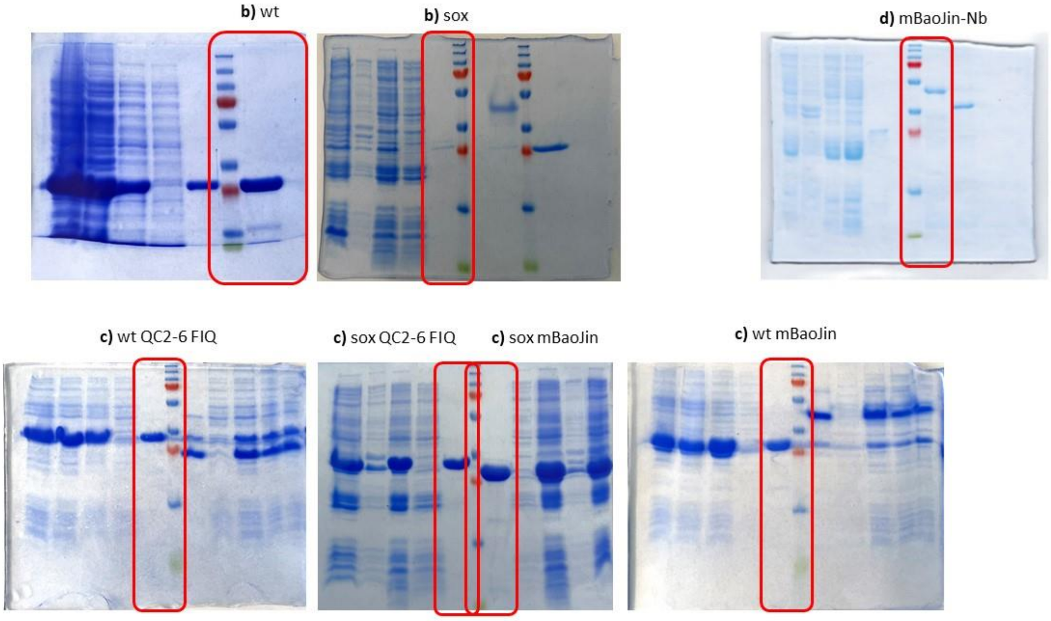

SF 1

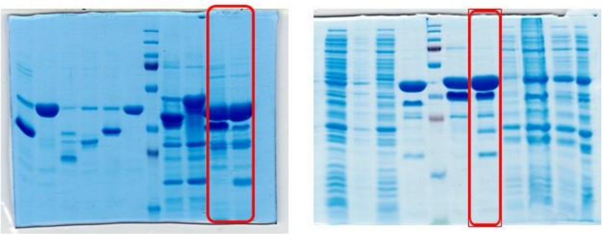

SF 2

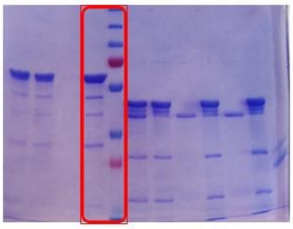

SF 3

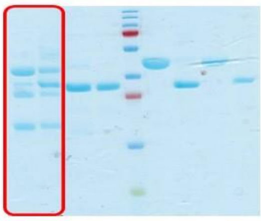

SF 4

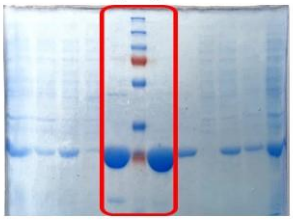

Supplement: Supplementary file 1 [file biomolecules-14-00587-s001.zip › biomolecules-2980957-supplementary.pdf]
